# Cimifugin Ameliorates Lipotoxicity-Induced Hepatocyte Damage and Steatosis through TLR4/p38 MAPK- and SIRT1-Involved Pathways

**DOI:** 10.1155/2022/4557532

**Published:** 2022-03-20

**Authors:** Wenwen Yang, Linwensi Zhu, Shanglei Lai, Qinchao Ding, Tiantian Xu, Rui Guo, Xiaobing Dou, Hui Chai, Zhiling Yu, Songtao Li

**Affiliations:** ^1^School of Public Health, Zhejiang Chinese Medical University, Hangzhou 310053, China; ^2^School of Life Science, Zhejiang Chinese Medical University, Hangzhou 310053, China; ^3^Academy of Chinese Medical Science, Zhejiang Chinese Medical University, Hangzhou, Zhejiang 310053, China; ^4^The First Affiliated Hospital of Zhejiang Chinese Medical University, Hangzhou 310053, China; ^5^Institute of Nutrition and Health, Zhejiang Chinese Medical University, Hangzhou 310053, China; ^6^School of Chinese Medicine, Hong Kong Baptist University, Hong Kong 999077, China

## Abstract

**Objective:**

Hepatic metabolic disorder induced by lipotoxicity plays a detrimental role in metabolic fatty liver disease pathogenesis. Cimifugin (Cim), a coumarin derivative extracted from the root of *Saposhnikovia divaricata*, possesses multiple biological properties against inflammation, allergy, and oxidative stress. However, limited study has addressed the hepatoprotective role of Cim. Here, we investigate the protective effect of Cim against lipotoxicity-induced cytotoxicity and steatosis in hepatocytes and clarify its potential mechanisms.

**Methods:**

AML-12, a nontransformed mouse hepatocyte cell line, was employed in this study. The cells were incubated with palmitate or oleate to imitate hepatotoxicity or steatosis model, respectively.

**Results:**

Cim significantly reversed palmitate-induced hepatocellular injury in a dose-dependent manner, accompanied by improvements in oxidative stress and mitochondrial damage. Cim pretreatment reversed palmitate-stimulated TLR4/p38 MAPK activation and SIRT1 reduction without affecting JNK, ERK1/2, and AMPK pathways. The hepatoprotective effects of Cim were abolished either through activating TLR4/p38 by their pharmacological agonists or genetical silencing SIRT1 via special siRNA, indicating a mechanistic involvement. Moreover, Cim treatment improved oleate-induced hepatocellular lipid accumulation, which could be blocked by either TLR4 stimulation or SIRT1 knockdown. We observed that SIRT1 was a potential target of TLR4 in palmitate-treated hepatocytes, since TLR4 agonist LPS aggravated, whereas TLR4 antagonist CLI-095 alleviated palmitate-decreased SIRT1 expression. SIRT1 knockdown did not affect palmitate-induced TLR4. In addition, TLR4 activation by LPS significantly abolished Cim-protected SIRT1 reduction induced by palmitate. These results collaboratively indicated that TLR4-regulated SIRT1 pathways was mechanistically involved in the protective effects of Cim against lipotoxicity.

**Conclusion:**

In brief, we demonstrate the protective effects of Cim against lipotoxicity-induced cell death and steatosis in hepatocytes. TLR4-regulated p38 MAPK and SIRT1 pathways are involved in Cim-protected hepatic lipotoxicity. Cim is a potential candidate for improving hepatic metabolic disorders mediated by lipotoxicity.

## 1. Introduction

Lipotoxicity is a hallmark of multiple noncommunicable metabolic diseases, including metabolic fatty liver disease, diabetes mellitus, and obesity [1]. When hepatocytes suffer from excessive lipid exposure, especially nonesterified lipids, such as long-chain saturated free fatty acids (LCSFAs), nonesterified cholesterol, sphingolipids, and ceramides [2], more intracellular lipids will accumulate and further lead to cell damage, which is termed as hepatolipotoxicity. Among those nonesterified lipids, palmitic acid (C16:0), one of the most abundant saturated free fatty acids in the human body and food, is highly used in metabolic liver disease research to mimic lipotoxicity-induced hepatocyte injury [3]. On the other hand, unsaturated fatty acids (USFAs) induce less hepatotoxicity compared to LCSFAs and tend to protect against LCSFA-induced cell death. However, they are more likely to cause hepatic steatosis. For example, oleic acid (C18:1), the most abundant monounsaturated fatty acid in the body, is widely used in *in vitro* model of hepatic steatosis [4].

In the past decades, more cellular mechanisms underlying lipotoxicity-induced liver injury have been illustrated [5, 6]. Endoplasmic reticulum (ER) stress and oxidative stress have been well-documented in palmitate-induced hepatocytes death [7]. Palmitate exposure induces mitochondrial dysfunction, accompanied by mitochondrial membrane potential (MMP) loss, excessive reactive oxygen species (ROS) production, and Bcl-2/Bax ratio decrease, which in turn triggers mitogen-activated protein kinase- (MAPK-) mediated programmed cell death [8]. MAPK inhibition prevents palmitate-induced cell death in various types of cells, including hepatocytes [9]. In addition, we previously reported that the activation of adenosine monophosphate-activated protein kinase (AMPK) and sirtuin 1 (SIRT1) contributes to the protection of palmitate-induced hepatotoxicity [10].

Recently, we identified toll-like receptor 4 (TLR4), a transmembrane protein expressed on the surface of cells, as a critical target in palmitate-induced hepatocyte death [11]. TLR4 has long been known as a fundamental immunoreceptor via recognizing pathogen-associated molecules, e.g., lipopolysaccharide (LPS). TLR4 can also be stimulated by endogenous ligands, such as LCSFAs, which share the same signaling pathway as LPS [12]. We previously reported that inhibiting TLR4 reversed palmitate-stimulated hepatocyte death and oleate-induced hepatic lipid deposition, respectively [13]. Liver-specific TLR4-nulled mice exhibited strong resistance to high-fat diet-induced liver injury and hepatic steatosis [14].

Several lines of evidence have demonstrated that lipotoxicity improvement is an effective strategy to treat or improve metabolic diseases [15, 16]. Although there is no approved hepatolipotoxic drug in the clinic, evidence emerged that phytochemicals from medicinal herbs own a vigorous capacity to ameliorate lipotoxicity [17]. Among those plant-derived compounds, cimifugin (Cim) is a coumarin derivative extracted from the root of *Saposhnikovia divaricata*, which is a traditional Chinese medical herb called Fang-feng (防风) with the usage history for the clinical therapy of allergy, rheumatism, headache, and convulsion, especially for allergic dermatitis and skin pruritus [18]. It has been known that Cim possesses a strong ability to prevent inflammation, allergy, and oxidative stress [19–22]. Cim administration inhibited allergic inflammation on epithelial cells of type 2 atopic dermatitis mice [20]. Cim intervention also ameliorated imiquimod-induced psoriasis by inhibiting oxidative stress and inflammation via NF-*κ*B/MAPK pathway in mice or keratinocytes [21]. Additionally, Cim treatment inhibited LPS-induced activation of the MAPK signaling pathway and improved the production of proinflammatory factors, including TNF-*α* and IL-1*β* [22]. Since oxidative stress and inflammation are hallmarks in lipotoxicity-induced hepatocytes, we proposed that Cim is a potential candidate for the treatment of lipotoxicity-induced hepatocyte dysfunction.

The present study was designed to investigate the protective effects of Cim against lipotoxicity-induced hepatic injury and steatosis. Our results demonstrated for the first time that Cim treatment effectively rescued lipotoxicity-caused oxidative stress, mitochondrial membrane potential (MMP) reduction, steatosis, and even cell death in hepatocytes. Further mechanistic study revealed that a TLR4-regulated p38 MAPK and SIRT1 pathway contributed to the beneficial role of Cim.

## 2. Material and Methods

### 2.1. Chemicals

Cim was purchased from Chengdu Herbpurify Co., Ltd. (Sichuan, China). LPS and U46619 were purchased from Selleck Chemicals (Houston, TX). Hoechst 33342, palmitic acid, and oleic acid were obtained from Sigma-Aldrich (St. Louis, MO). LSFA-BSA conjugates were prepared as described previously [23]. All experiments included a control group/vehicle, which was exposed to an equal amount of solvent (e.g., BSA and DMSO as relevant).

### 2.2. Cell Culture

AML-12 mouse hepatocyte, a nontransformed cell line, was obtained from American Type Culture Collection (ATCC, Manassas, VA). AML-12 hepatocytes were cultured in Dulbecco's Modified Eagle Medium/Ham's Nutrient Mixture F-12 (DMEM/F12, 1 : 1, Hyclone) containing 10% (*v*/*v*) fetal bovine serum (FBS, Biological Industries, ISR), 5 mg/mL insulin (Solarbio, Beijing, China), 5 *μ*g/mL transferrin (Solarbio, Beijing, China), 5 ng/mL selenium (Sigma-Aldrich, St. Louis, MO), and 40 ng/mL dexamethasone (Sigma-Aldrich, St. Louis, MO) at 37°C in a humidified atmosphere of 5% CO_2_ and 95% air.

### 2.3. Cell Death Assay

Hepatocyte injury was determined by MTT test, lactate dehydrogenase (LDH) release in the cultured medium, and nuclear staining as described previously [24], respectively. For the LDH assay, the culture medium was collected and detected using an LDH assay kit (Thermo Scientific Inc., VA) according to the manufacturer's instructions. In Hoechst staining, the cells were stained with Hoechst 33342 staining solution (5 mg/L) for 10 min. After PBS rinsing, the cells were imaged by fluorescent microscope (Leica, Wetzlar, Germany). For the MTT test, the cells were seeded in a 96-well plate at a density of 2 × 10^4^/well and cultured to 80% confluence. After indicated treatments, 20 *μ*L of fresh MTT (3-(4, 5-dimethylthiazol-2-yl)-2, 5-diphenyltetrazolium bromide, 5 mg/mL) was added into each well. The cells were incubated at 37°C for 4 hours to allow incorporation and conversion of MTT to formazan derivative. The formazan derivative was solubilized by DMSO. After incubation for 10 minutes at room temperature on the rocker, the absorbance values were measured at 470 nm using FLUOstar Omega (BMG Labtech, Offenburg, Germany).

### 2.4. ROS Detection

Intracellular ROS was measured as previously described [25]. Briefly, after the indicated treatments, the cells were washed and placed in a serum-free medium. 2,7-Dichlorodi-hydrofluorescein diacetate (DCFH-DA) was added to each well at a final concentration of 10 *μ*M. At the completion of the incubation, the cells were washed three times with ice-cold phosphate-buffered saline (PBS), and then, the fluorescence was measured by an inverted fluorescent microscope (Leica, Wetzlar, Germany). Mean fluorescence intensity (MFI) from five random fields was analyzed using ImageJ 1.41 software.

### 2.5. MMP Assay

MMP was measured using the fluorescent cell-permeable dye Rh123. Rh123 (100 *μ*g/mL) was added to the medium for 45 min at 37°C. Fluorescence was measured using a fluorescent microscope (Leica, Wetzlar, Germany). The MFI from five random fields was analyzed with ImageJ 1.41 software.

### 2.6. Lipid Deposition Assay

Intracellular lipid deposition was determined by the measurement of triglyceride (TG) and lipid staining. For intracellular TG detection, the cells were collected and lysed.

TG and protein concentration were determined by TG assay kit (Nanjing Jiancheng Bioengineering Institute, Nanjing, China) and BCA kit (Beyotime, Shanghai, China), respectively, according to the manufacturer's instructions. The ratio of TG level to protein concentration was calculated to express the relative TG content in the cells.

### 2.7. Quantitative Real-Time PCR

Total RNA was extracted from cells by TRIzol method (Invitrogen, CA). The quality of RNA was determined by NanoDrop (Thermo Scientific, MA). Amplification of the corresponding genes was performed by StepOnePlus Real-Time PCR System (Applied Biosystems, Foster City, CA, USA). The data were calculated by 2^-(△△CT)^ and analyzed for fold induction of each gene as compared with the blank sample. The primers used are shown in Table 1.

### 2.8. Western Blot Analysis

Western blots were performed as described previously [26] to determine changes in protein content and/or phosphorylation. The following antibodies were used: anti-cleaved-caspase3, anti-Bcl2, anti-Bax, anti-phospho-JNK, anti-JNK, anti-phospho-p38, anti-p38, anti-ERK1/2, anti-phospho-ERK1/2, anti-AMPK, anti-phospho-AMPK, anti-TLR4, anti-SIRT1, and anti-GAPDH from Cell Signaling Technology Inc. (Beverly, MA) and anti-TLR4 from Santa Cruz Biotechnology (Santa Cruz, CA), and GAPDH is used as an internal control.

### 2.9. RNA Interference

Small interfering RNA (siRNA) for mouse SIRT1 was purchased from GenePharma Co., Ltd. (Shanghai, China). SiRNA-Mate (GenePharma, Shanghai, China) was utilized to deliver siRNA to the targeted cells according to the manufacturer's protocol. Scrambled siRNA (GenePharma, Shanghai, China) was applied in negative control group. Silencing efficiency was verified by western blot analysis.

### 2.10. Statistical Analyses

All data were expressed as means ± SD of at least three independent biological experiments with three replicates in each experiment. Statistical analyses were carried out using SPSS 16.0 software. The differences between treatments were performed using a one-way ANOVA and analyzed by post hoc test with Fisher's least significant difference (LSD). Differences between treatments were considered to be statistically significant at *P* < 0.05.

## 3. Results

### 3.1. Cim Prevents Hepatocytes against Palmitate-Induced Cell Death

**Figure 1:**
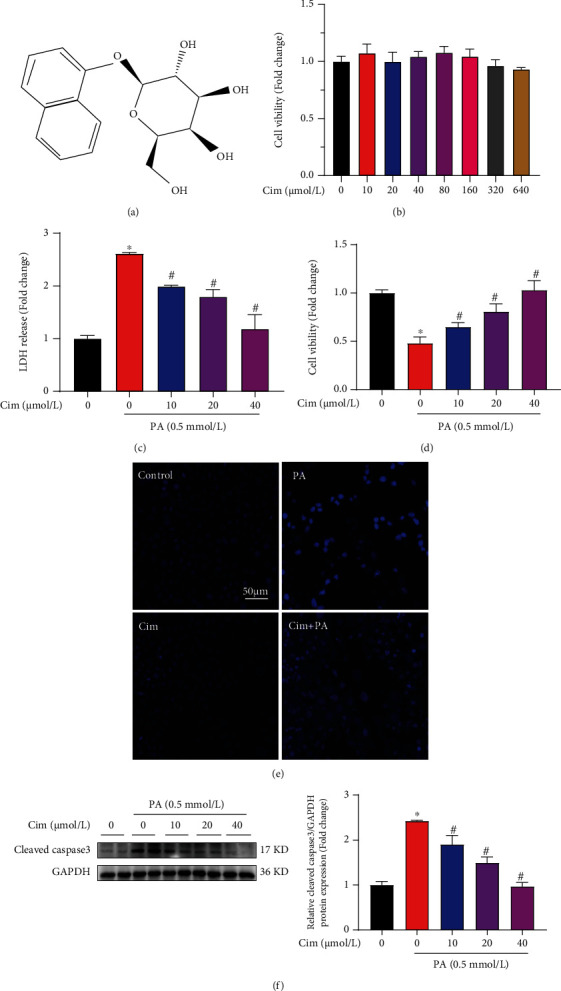
Cimifugin protects hepatocytes against palmitate-induced cell death. (a) Chemical structural formula of cimifugin (Cim). (b) AML-12 mouse hepatocytes were treated with different doses (0, 10, 20, 40, 80, 160, 320, and 640 *μ*M) of Cim. MTT assay was performed to test cell viability after 24 h incubation. The protective role of Cim against lipotoxicity was performed as follow: AML-12 hepatocytes were exposed to palmitic acid (PA, 0.5 mM) for 16 h. Cim (40 *μ*M or as indicated doses) was added 2 h before palmitate treatment. Cell injury was evaluated by (c) LDH release, (d) MTT test, (e) nuclear staining, and (f) cleaved-caspase3 expression, respectively. All values are denoted as means ± SD from at least three independent batches of cells. ∗ and # reflect statistical difference (*P* < 0.05) compared with the control group and palmitate treatment group, respectively.

The chemical structure formula of Cim is shown in Figure 1(a). We firstly evaluated safe dose of Cim on hepatocytes by MTT test and observed that Cim treatment did not induce any cytotoxicity at the dosage up to 640 *μ*M in AML-12 hepatocytes (Figure 1(b)). The protective effect of Cim against palmitate-induced cell death was measured by both LDH release and MTT test. Our data indicated that Cim pretreatment significantly reversed palmitate-induced hepatocytes death in a dose-dependent manner (Figures 1(c) and 1(d)). Cim also rescued palmitate-stimulated injury on nuclear chromatin in AML-12 hepatocytes (Figure 1(e)). Moreover, Cim markedly improved palmitate-caused activation of cleaved-caspase-3, which is a typical marker of apoptosis (Figure 1(f)). To further corroborate our observations, similar experiments were carried out in HepG2 cells (human hepatoma cells). Our data showed that palmitate-induced cell death in human hepatocytes was also significantly inhibited by Cim pretreatment (Supplementary data, Fig. S1). These results indicated that Cim intervention protected hepatocytes from palmitate-induced injury through antiapoptotic relative mechanism(s).

### 3.2. Cim Alleviates Palmitate-Induced Hepatic Mitochondrial Dysfunction

**Figure 2:**
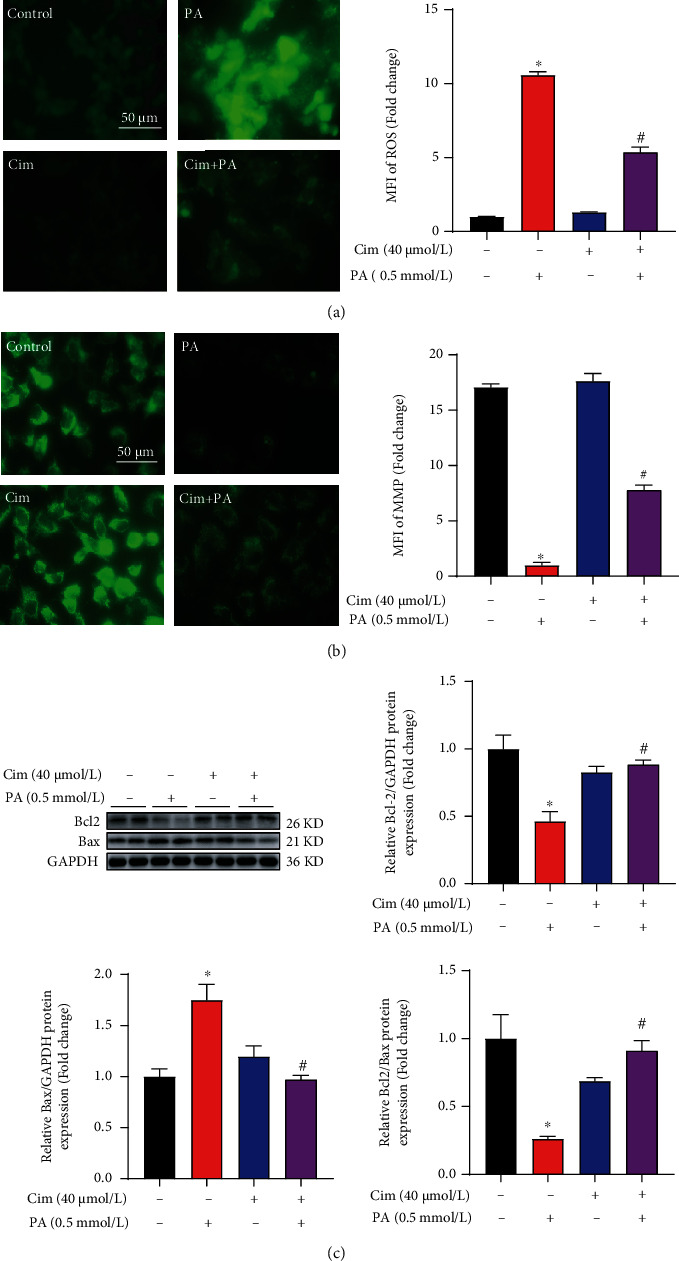
Cimifugin improves palmitate-induced mitochondrial dysfunction in hepatocytes. AML-12 hepatocytes were treated with palmitic acid (PA, 0.5 mM) for 16 h with or without cimifugin (Cim, 40 *μ*M) pretreatment for 2 h. (a) Intracellular reactive oxygen species (ROS) was detected by DCFH-DA staining. (b) Mitochondrial membrane potential (MMP) was analyzed by Rh123 staining. (c) Bcl-2 and Bax expressions were measured by immunoblotting. All values are denoted as means ± SD from at least three independent batches of cells. ∗ and # reflect statistical difference (*P* < 0.05) compared with the control group and palmitate treatment group, respectively.

Mitochondrion, a vital organelle for fatty acid catabolism, plays a critical role in the regulation of intracellular redox homeostasis. When hepatocytes are exposed to excessive LSFAs, more ROS were generated and escaped from mitochondria, which further led to cell dysfunction or even mitochondrial-programmed apoptosis [13]. In this study, we observed that palmitate exposure significantly increased intracellular ROS accumulation in AML-12 hepatocytes, which was significantly ameliorated by Cim pretreatment (Figure 2(a)). MMP, an indicative marker of mitochondria function, was reduced by palmitate exposure (Figure 2(b)). Cim preincubation significantly reversed palmitate-induced hepatic MMP loss (Figure 2(b)). Moreover, palmitate-caused adverse changes in Bcl-2 and Bax expression and Bcl2/Bax ratio were markedly rescued by Cim preintervention (Figure 2(c)). These results implied that defending mitochondrial-driven apoptosis via antioxidative stress-mediated mechanism(s) might be involved in the protective role of Cim.

### 3.3. TLR4 Contributes to Cim-Inhibited Hepatic Lipotoxicity

**Figure 3:**
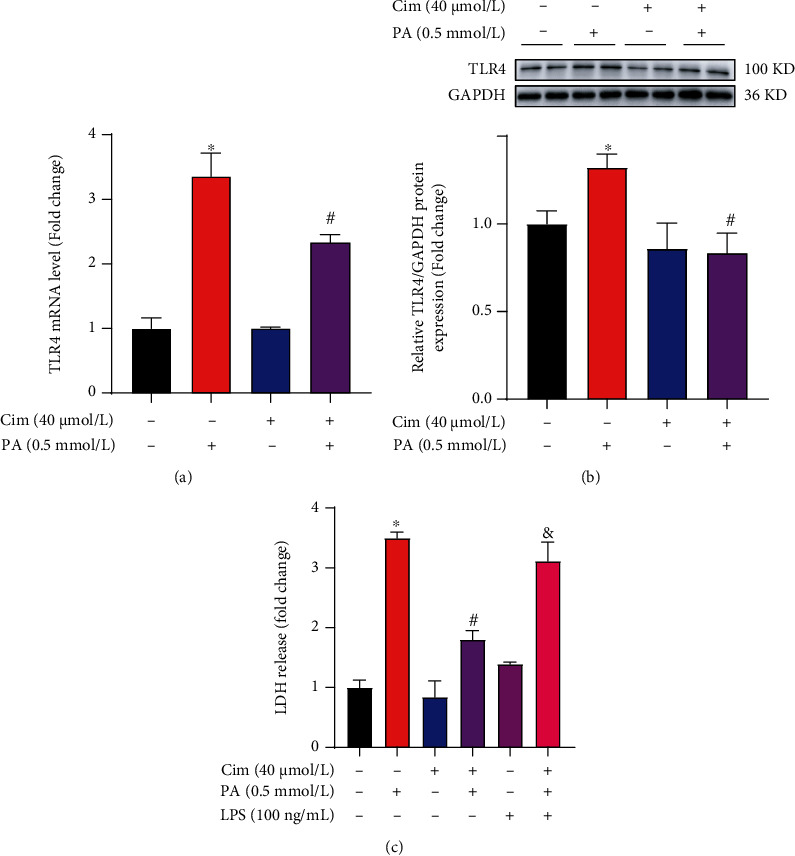
TLR4 reduction contributes to cimifugin-protected hepatic lipotoxicity. AML-12 hepatocytes were treated with palmitic acid (PA, 0.5 mM) for 16 h with or without cimifugin (Cim, 40 *μ*M) pretreatment for 2 h. LPS (100 ng/mL) was added 1 h before Cim treatment. (a) mRNA of *TLR4*. (b) Protein expression of TLR4. (c) Cell death was evaluated by LDH release in the cultured medium. All values are denoted as means ± SD from at least three independent batches of cells. ∗, #, and & reflect statistical difference (*P* < 0.05) compared with the control group, palmitate treatment group, and PA+Cim group, respectively.

We previously reported that TLR4, a critical regulator in inflammatory signaling pathway, was involved in palmitate-induced cell death in AML-12 hepatocytes [27]. Considering the anti-inflammatory effect of Cim, we speculated whether TLR4 contributes to Cim-inhibited hepatic lipotoxicity. Our results indicated that pretreating hepatocytes with Cim significantly reduced palmitate-stimulated TLR4 upregulation at both transcriptional and protein levels (Figures 3(a) and 3(b)). To further confirm this observation, we conducted the similar test in HepG2 cells. Our data showed that palmitate-induced TLR4 upregulation was also significantly blocked by Cim pretreatment (Supplementary data, Fig. S2). Moreover, TLR4 induction by its special agonist LPS significantly abolished Cim-protected lipotoxicity (Figure 3(c)), indicating the involvement of the TLR4 pathway in the beneficial role of Cim.

### 3.4. Cim Improves Palmitate-Induced p38 MAPK Pathway

**Figure 4:**
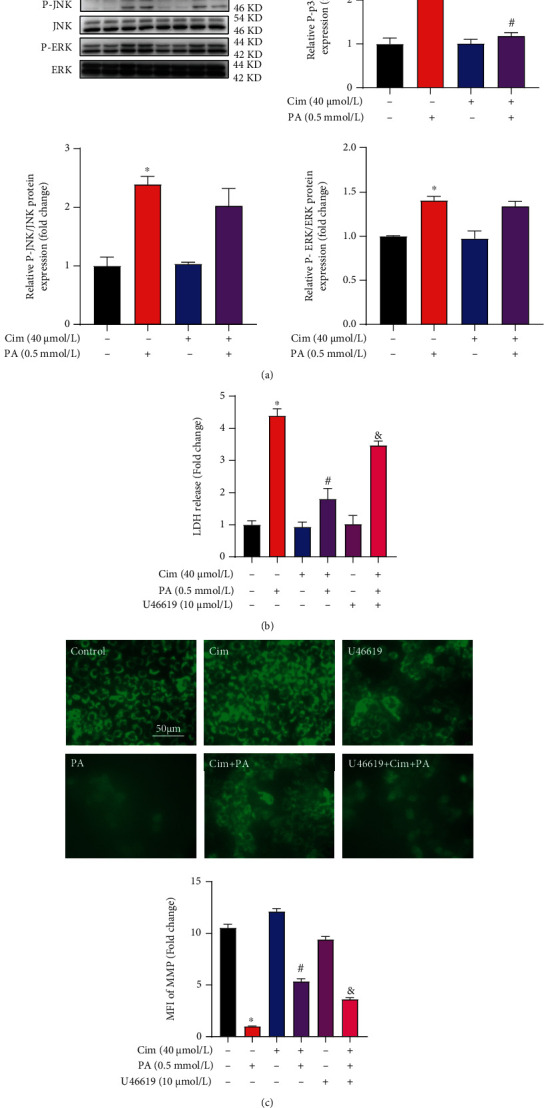
p38 inhibition is involved in cimifugin-protected hepatic lipotoxicity. AML-12 hepatocytes were treated with palmitic acid (PA, 0.5 mM) for 16 h with or without cimifugin (Cim, 40 *μ*M) pretreatment for 2 h. U46619 (10 *μ*M) was added 1 h before Cim treatment. (a) The expressions of phosphorylated-p38, -JNK, and -ERK1/2 were determined by immunoblotting. (b) Cell death was evaluated by LDH release in the cultured medium. (c) Mitochondrial membrane potential (MMP) was analyzed by Rh123 staining. All values are denoted as means ± SD from at least three independent batches of cells. ∗, #, and & reflect statistical difference (*P* < 0.05) compared with the control group, palmitate treatment group, and PA+Cim group, respectively.

Evidence has implicated that MAPK pathway, which has been recognized as the target of TLR4 [11], was involved in palmitate-induced hepatocyte death. We therefore explored whether MAPK pathway is involved in Cim-alleviated lipotoxicity. Our data showed that palmitate incubation stimulated phosphorylation of p38, JNK, and ERK1/2 (Figure 4(a)), while Cim pretreatment only reversed phosphorylated-p38 induction by palmitate (Figure 4(a)). Moreover, p38 activation by its pharmacal agonist U46619 significantly blocked the protective role of Cim on palmitate-induced cell death and MMP loss (Figures 4(b) and 4(c)). These results indicated that the inhibition of p38 MAPK contributed to the beneficial effect of Cim.

### 3.5. SIRT1 Is Involved in Cim-Protected Hepatic Lipotoxicity

**Figure 5:**
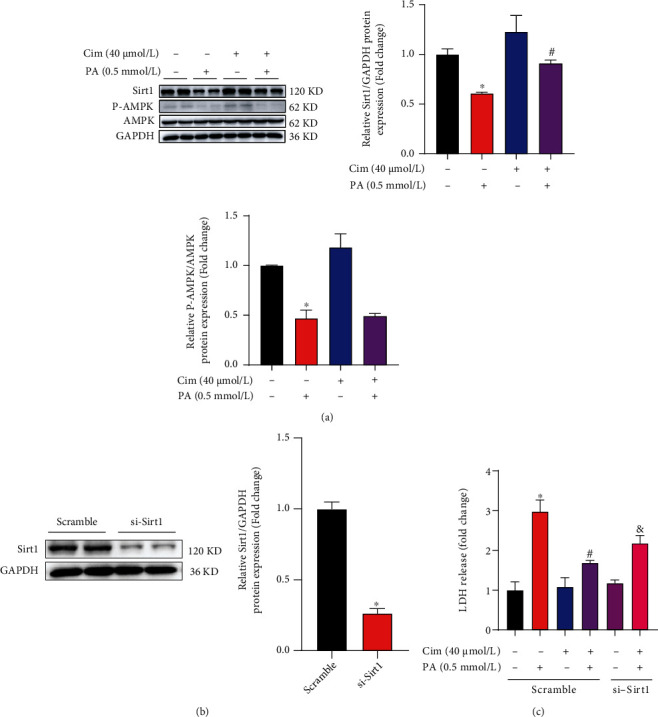
SIRT1 upregulation participates in cimifugin-protected hepatic lipotoxicity. AML-12 hepatocytes were treated with palmitic acid (PA, 0.5 mM) for 16 h with or without cimifugin (Cim, 40 *μ*M) pretreatment for 2 h. For SIRT1 silencing, AML-12 cells were transfected with si-SIRT1 or scramble siRNA before Cim intervention. (a) The expressions of SIRT1 and phosphorylated-AMPK were determined by immunoblotting. (b) SIRT1 silencing efficiency was verified by protein expression. (c) Cell death was evaluated by LDH release in the cultured medium. All values are denoted as means ± SD from at least three independent batches of cells. ∗, #, and & reflect statistical difference (*P* < 0.05) compared with the control group, palmitate treatment group, and PA+Cim group, respectively.

We previously reported that the inhibition of both SIRT1 and AMPK was mechanistically involved in palmitate-induced hepatotoxicity [28]. Therefore, we also tested the involvement of SIRT1 and AMPK in Cim-protected hepatic lipotoxicity in this study. Our data showed that palmitate exposure markedly inhibited expression of SIRT1 and phosphorylated-AMPK, whereas Cim pretreatment only reversed SIRT1 reduction, but not phosphorylated-AMPK (Figure 5(a)). The similar result was also observed in HepG2 cells that Cim preincubation prevented palmitate-decreased SIRT1 expression (Supplementary data, Fig. S2). Importantly, genetically silencing SIRT1 expression using its special siRNA (Figure 5(b)) significantly blocked Cim-protected lipotoxicity (Figure 5(c)). These results demonstrated that SIRT1 was mechanistically in the protective role of Cim against palmitate-induced hepatic cell death.

### 3.6. Cim Ameliorates Oleate-Induced Steatosis in Hepatocytes

**Figure 6:**
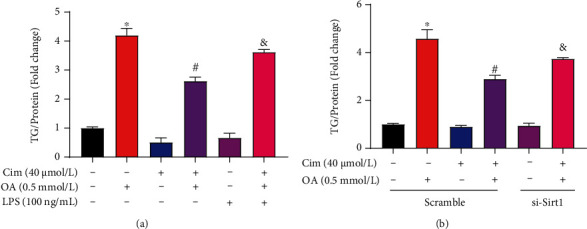
Cimifugin ameliorates oleate-induced steatosis in hepatocytes. AML-12 hepatocytes were treated with oleic acid (OA, 0.5 mM) for 16 h with or without cimifugin (Cim, 40 *μ*M) pretreatment for 2 h. LPS (100 ng/mL) was added 1 h before Cim treatment. For SIRT1 silencing, AML-12 cells were transfected with si-SIRT1 or scramble siRNA before Cim intervention. (a and b) Intracellular triglyceride (TG) was measured as described in Material and Methods. All values are denoted as means ± SD from at least three independent batches of cells. ∗, #, and & reflect statistical difference (*P* < 0.05) compared with the control group, palmitate treatment group, and PA+Cim group, respectively.

Subsequently, we evaluated antisteatotic role of Cim by measuring the lipid accumulation in AML-12 hepatocytes. Our data showed that oleate exposure significantly elevated lipid accumulation in AML-12 and HepG2 hepatocytes, respectively, while Cim pretreatment significantly decreased oleate-caused lipid deposition (Figure 6(a) and Supplementary data, Fig. S3). In addition, Cim preincubation also protected palmitate-induced lipid accumulation in hepatocytes (Supplementary data, Fig. S4). Preincubating hepatocytes with LPS, a chemical agonist of TLR4, markedly inhibited Cim-improved lipid accumulation in AML-12 hepatocytes (Figure 6(a)). Moreover, genetically knocking down SIRT1 significantly blocked Cim-protected lipid deposition (Figure 6(b)). Those results indicated that the lipid-lowering effects of Cim in hepatic steatosis may be mediated by TLR4- and SIRT1-dependent pathways.

### 3.7. TLR4 and SIRT1 Pathways Are Independent of Each Other in Hepatocytes

**Figure 7:**
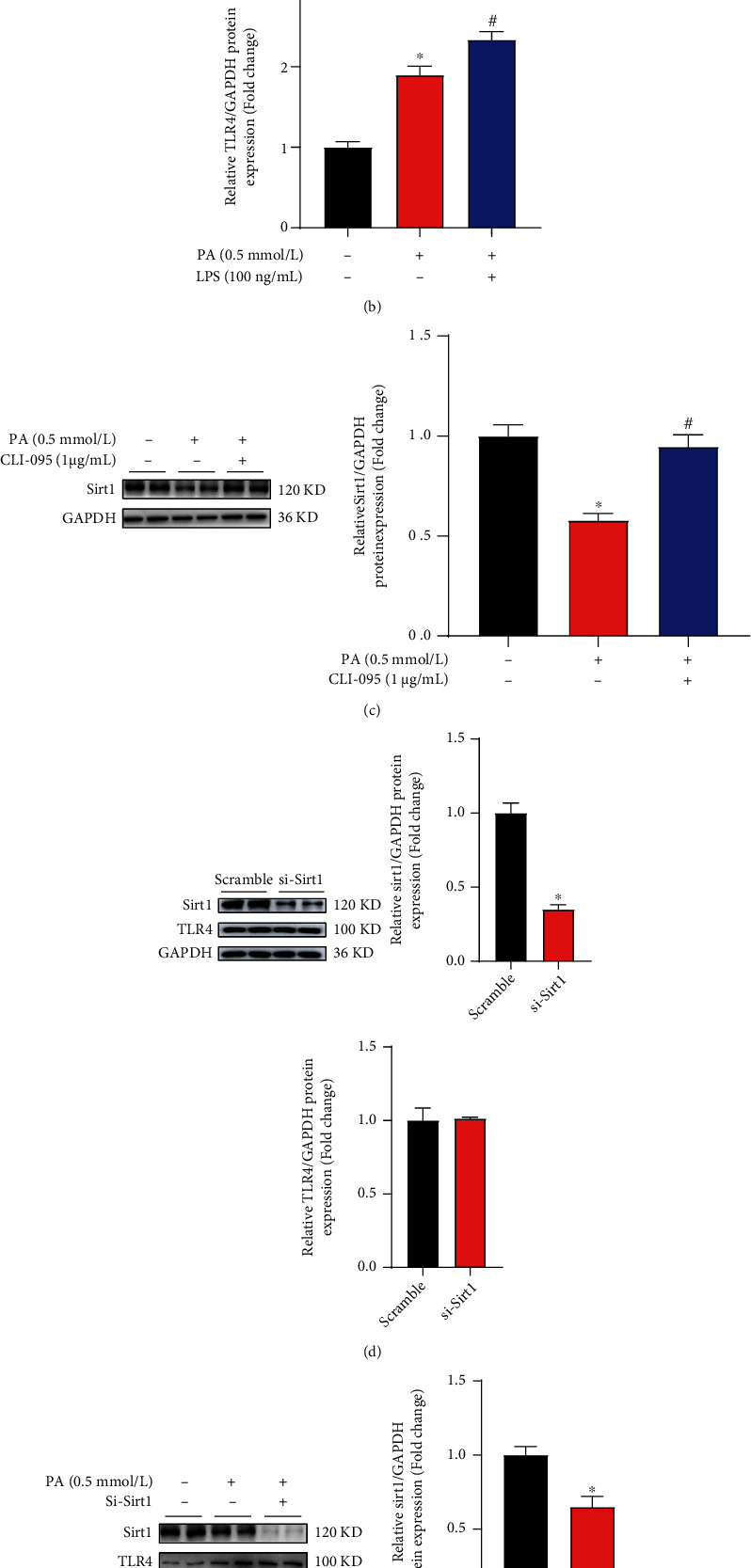
TLR4-regulated SIRT1 contributes to cimifugin-protected lipotoxicity. (a) AML-12 hepatocytes were treated with LPS (100 ng/mL). The expressions of TLR4 and SIRT1 were detected. (b and c) AML-12 hepatocytes were treated with palmitic acid (PA, 0.5 mM) for 16 h. LPS (100 ng/mL) or CLI-095 (1 *μ*g/mL) was added 1 h before PA treatment, respectively. TLR4 and SIRT1 expressions were detected. (d) AML-12 cells were transfected with si-SIRT1 or scramble siRNA. After 16 h transfection, the expressions of TLR4 and SIRT1 were detected. (e) After si-SIRT1 transfection, PA (0.5 mM) was added for 16 h. TLR4 and SIRT1 were detected. (f) AML-12 cells were treated with palmitic acid (PA, 0.5 mM) for 16 h with or without cimifugin (Cim, 40 *μ*M) pretreatment for 2 h. LPS (100 ng/mL) was added 1 h before Cim treatment. SIRT1 expression was detected. All values are denoted as means ± SD from at least three independent batches of cells. ∗, #, and & reflect statistical difference (*P* < 0.05) compared with the control group, palmitate treatment group, and PA+Cim group, respectively.

Several studies have reported the reduction of SIRT1 expression in LPS-treated mice liver and cultured macrophages [29, 30]. Since LPS is a well-known agonist of TLR4, we then speculated that SIRT1 was a potential downstream target of TLR4 in response to lipotoxicity in hepatocytes and tested whether there existed a TLR4/SIRT1 pathway contributing to Cim-protected hepatic lipotoxicity. Our data indicated that TLR4 activation by LPS per se was negatively associated with SIRT1 reduction (Figure 7(a)). However, in the presence of palmitate, LPS induced more reduction of SIRT1 than that in palmitate-treated cells (Figure 7(b)). Inhibiting TLR4 using its special antagonist (CLI-095) significantly abolished PA-decreased SIRT1 expression (Figure 7(c)). Besides, SIRT1 knockdown neither induced TLR4 upregulation nor enhanced palmitate-induced TLR4 upregulation (Figures 7(d) and 7(e)). Those results collectively indicated that SIRT1 is a potential down-target in palmitate-stimulated TLR4. In addition, TLR4 activation by LPS significantly blocked Cim-increased SIRT1 in the presence of palmitate (Figure 7(f)), suggesting that a potential TLR4/SIRT1 pathway was mechanistically involved in the protective role of Cim.

## 4. Discussion

In the present study, we reported for the first time that Cim exerts a strong preventive effect against lipotoxicity-induced cell death and steatosis in AML-12 hepatocytes. Further mechanistic investigations demonstrated that TLR4/p38 and SIRT1 signaling pathways were involved in Cim-protected lipotoxicity.

Lipotoxicity originated from abnormal lipid metabolism played a seriously detrimental role in metabolic diseases, including fatty liver diseases, obesity, cardiovascular diseases, and diabetes. Several lines of evidence indicated that alleviating lipotoxicity was an effective strategy for preventing or improving multiple metabolic diseases [31, 32]. However, no medication targeting lipotoxicity has been proved in clinics. Previous studies, including ours, have demonstrated that phytochemicals extracted from plant foods or medicinal herbs are potential effective candidates for preventing lipotoxicity [33]. Cim is the main pharmacodynamic component of *Saposhnikovia divaricate*, which is a traditional Chinese medical herb (termed as Fang-feng in Chinese) and widely used for antipyretic, analgesic, and anti-inflammatory purposes. Besides Cim, prim-o-glucosylcimifugin is another ingredient in Fang-feng, which can be metabolized into Cim *in vivo* [19]. Several studies have reported the anti-inflammatory and antioxidative stress roles of Cim [19–22]. Considering inflammation and oxidative stress are hallmarks in lipotoxicity-induced cell death, we wondered whether Cim could protect hepatocytes from lipotoxicity.

A limited study has reported lipotoxicity protective role of Cim before. Firstly, we analyzed the cytotoxic effect of Cim on AML-12 hepatocytes. We did not observe any cytotoxic effect of Cim on hepatocytes at a dose as high as 640 *μ*M. In agreement with our observation, Han et al. also reported that 100 mg/L (326.47 *μ*M) Cim did not cause cytotoxicity in RAW264.7 cells after 72 h incubation [22]. These results proved that Cim at this dosage is biologically safe. The selected dosage of Cim in this study was based on the careful considerations of the clinical prescription dosage of Fang-feng, the content of Cim in Fang-feng, and the pharmacokinetics of water extract of *Saposhnikovia divaricate*. The content of Cim in Fang-feng water extract is about 2.48 mg/g [34]. The common usage dose of Fang-feng in the clinical prescription is about 10-30 g/d. It means that the theoretical intake of Cim is about 24.8-74.4 mg/d. Without considering the absorption, the ideal peak concentration in the human body will be about 20.2-60.7 *μ*M. According to the pharmacokinetic performance of Fang-feng in experimental animals [34], the estimated peak concentration of Cim in human blood will be 7.3-21.9 *μ*M, which was calculated based on body weight and blood volume. Besides, 50 *μ*M of Cim was selected in a previous *in vitro* study [19]. Taken those evidence together, a dose of Cim ranged from 10 *μ*M to 40 *μ*M was chosen in the present study. Consistent with our hypotheses, our data clearly showed that Cim intervention significantly improved cell death and lipid deposition induced by lipotoxicity.

Lipotoxicity-induced hepatocytes injury is characterized by mitochondria dysfunction. When mitochondria are suffered from excessive LSFAs, imbalanced redox homeostasis, accompanied by more intracellular ROS generation and MMP loss, can occur. Meanwhile, the mitochondria apoptosis-related proteins, such as antiapoptotic member Bcl-2 and proapoptotic protein Bax, which controls the permeabilization of the mitochondrial membrane, are critical targets in response to lipotoxicity via upregulating cleaved-caspase-3 activation [19]. We previously reported palmitate-induced ROS production, impaired MMP, and decreased Bcl2/Bax ratio in AML-12 hepatocytes [35]. However, limited study has addressed the protective role of Cim on lipotoxicity-induced mitochondria dysfunction. In this study, we provided clear evidence that Cim intervention protected hepatocytes from lipotoxicity-induced mitochondria damage.

Emerging evidence, including ours, showed that TLR4 was a key target in lipotoxicity-induced hepatic injury [24]. In high-fat diet-induced obese mice, liver TLR4 expression was significantly stimulated [11], while liver-specific knockout TLR4 rescued high-fat diet-induced liver injury and steatosis [36]. We recently reported that inhibiting TLR4 alleviated oleate-induced lipid accumulation in AML-12 hepatocytes [13]. Considering the proinflammatory role of TLR4 and the anti-inflammatory effect of Cim, we presumed that TLR4 might be a potential target in Cim-protected lipotoxicity. None of the published article has reported the regulatory role of Cim on TLR4 expression before our study. Here, we observed that Cim treatment significantly reduced palmitate-activated TLR4 at both transcriptional and protein levels. At the same time, activating TLR4 by its agonist (LPS) markedly abolished the beneficial role of Cim on lipotoxicity-induced hepatic cell death and steatosis. These data implied that TLR4 was mechanistically involved in Cim-alleviated lipotoxicity. However, the present study did not provide direct evidence on how Cim preincubation prevents palmitate-induced TLR4 activation. To the best known of our knowledge, few studies have addressed the reasons through which palmitate can stimulate TLR4. In this study, we observed that Cim pretreatment significantly improved palmitate-induced increase of TLR4 mRNA, implying the involvement of a transcriptional mechanism. Several studies have demonstrated that TLR4 could be transcriptionally regulated by microRNAs, including miR-26a, miR-140-5p, miR-124, miR-27a, and miR-17 in various types of cells [37–41]. Besides, we also searched the TLR4 promoter sequence (NC_000070.7, -2000~100) in the websites including JASPAR (https://jaspar.genereg.net/) and GeneCards database (http://www.genecards.org) and obtained 6 (including YY1, JUND, SPI1, IKZF1, ETV6, and NR2F2) coexisting nuclear factors in these databases (Supplementary data, Fig. S5). Further study should be conducted to address how Cim protects transcriptional activation in palmitate-induced TLR4 based on the clues listed above.

As the well-known targets of TLR4, the participation of MAPK pathway was subsequently considered in this study. Several lines of evidence demonstrated that MAPKs, including JNK, p38, and ERK1/2, were activated in both high-fat diet-fed mice liver and LSFA-exposed cells [42]. In hepatocytes, we previously reported that the inhibition of either p38 or JNK MAPK prevented palmitate-induced cell death [43]. Besides, MAPK inhibition also improved liver injury and steatosis induced by high-fat diet consumption [44]. In this study, we confirmed that the p38 MAPK pathway was involved in Cim-protected lipotoxicity, evidenced by the fact that Cim significantly reversed palmitate-stimulated phosphorylated-p38 upregulation and p38 agonist (U46619) blocked the beneficial role of Cim. Those results were agreed with the previous evidence that Cim ameliorated other stimuli-triggered activation of MAPKs in both experimental animals and cultured cells [22, 45].

Recently, we reported that the activation of AMPK-regulated SIRT1 pathway prevented lipotoxicity-induced hepatocytes death [10]. Since a limited study has evaluated the regulative role of Cim on AMPK and SIRT1 before, we investigated the involvement of AMPK and SIRT1 pathways in Cim-protected lipotoxicity in this study. We observed that Cim treatment significantly rescued palmitate-induced SIRT1 reduction without affecting palmitate-inhibited AMPK phosphorylation. Importantly, genetically knocking down SIRT1 blocked the protective role of Cim on both palmitate-induced hepatocytes death and oleate-stimulated lipid accumulation, which implied that SIRT1 was mechanistically involved in the beneficial role of Cim. Previous studies revealed that SIRT1 could be downregulated by LPS, a commonly used agonist of TLR4 [28, 29]. Therefore, we speculated that lipotoxicity-induced SIRT1 reduction was positively associated with TLR4 activation since LCSFAs could function as an endogenous ligand of TLR4. Our results clearly indicated that TLR4-regulated SIRT1 was participated in mechanisms of Cim-protected lipotoxicity. Further studies are still needed to clarify how TLR4 regulates SIRT1 in hepatocytes and whether Cim supplementation could alleviate high-fat diet-induced hepatic steatosis and liver injury.

In summary, our study provides strong evidence for the first time that Cim intervention prevents lipotoxicity-induced cell death and steatosis in hepatocytes via inhibiting TLR4/p38 overactivation and SIRT1 reduction. We highlighted the potential value of Cim as an effective candidate in preventing and/or treating liver diseases with lipotoxicity as a critical pathological feature.

## Figures and Tables

**Table 1:** Primer sequence for quantitative real-time PCR.

Gene		Primer sequence (5′-3′)	Products size (bp)
*TLR4*	Forward reverse	AAATGCACTGAGCTTTAGTGGT TGGCACTCATAATGATGGCAC	104
*18*s	Forward reverse	GAATGGGGTTCAACGGGTTA AGGTCTGTGATGCCCTTAGA	109

## Data Availability

Readers can access the data supporting the conclusions of the study from the corresponding author Songtao Li.

## Supplementary Materials

Supplementary Materials Figure S1: HepG2, a widely used human hepatoma cell line, was obtained from American Type Culture Collection (ATCC, Manassas, VA). HepG2 hepatocytes were cultured in Dulbecco's Modified Eagle Medium (DMEM, Gibco) containing 10% (*v*/*v*) fetal bovine serum (FBS, Biological Industries) at 37°C in a humidified atmosphere of 5% CO_2_ and 95% air. HepG2 hepatocytes were treated with palmitic acid (PA, 0.5 mM) for 16 h with or without cimifugin (Cim, 40 *μ*M) pretreatment for 2 h. Intracellular triglyceride (TG) was measured as described in Material and Methods. All values are denoted as means ± SD from at least three independent batches of cells. ∗ and # reflect statistical difference (*P* < 0.05) compared with the control group and palmitate treatment group, respectively. Figure S2: the source and culture method of HepG2 hepatocytes were same as those of Figure S1. HepG2 hepatocytes were treated with palmitic acid (PA, 0.5 mM) for 16 h with or without cimifugin (Cim, 40 *μ*M) pretreatment for 2 h. SIRT1 and TLR4 expressions were measured by immunoblotting. ∗ and # reflect statistical difference (*P* < 0.05) compared with the control group and palmitate treatment group, respectively. Figure S3: the source and culture method of HepG2 hepatocytes were same as those of Figure S1. HepG2 hepatocytes were treated with oleic acid (OA, 0.5 mM) for 16 h with or without cimifugin (Cim, 40 *μ*M) pretreatment for 2 h. Intracellular triglyceride (TG) was measured as described in Material and Methods. ∗ and # reflect statistical difference (*P* < 0.05) compared with the control group and palmitate treatment group, respectively. Figure S4: the source and culture method of HepG2 hepatocytes were same as those of Figure S1. HepG2 hepatocytes were treated with palmitic acid (PA, 0.5 mM) for 16 h with or without cimifugin (Cim, 40 *μ*M) pretreatment for 2 h. Intracellular triglyceride (TG) was measured as described in Material and Methods. ∗ and # reflect statistical difference (*P* < 0.05) compared with the control group and palmitate treatment group, respectively. Figure S5: the TLR4 promoter sequence (NC_000070.7, -2000~100) was searched in the websites including JASPAR (https://jaspar.genereg.net/) and GeneCards database (http://www.genecards.org), and the coexisting nuclear factors were obtained in these databases.
