# Supplementary material for: Cimifugin Ameliorates Lipotoxicity-Induced Hepatocyte Damage and Steatosis through TLR4/p38 MAPK- and SIRT1-Involved Pathways
Source: Oxid Med Cell Longev. 2022 Mar 20;2022:4557532. doi: 10.1155/2022/4557532 (PMC8958062; doi:10.1155/2022/4557532)
Supplement: Supplementary Materials — Figure S1: HepG2, a widely used human hepatoma cell line, was obtained from American Type Culture Collection (ATCC, Manassas, VA). HepG2 hepatocytes were cultured in Dulbecco's Modified Eagle Medium (DMEM, Gibco) containing 10% (v/v) fetal bovine serum (FBS, Biological Industries) at 37°C in a humidified atmosphere of 5% CO2 and 95% air. HepG2 hepatocytes were treated with palmitic acid (PA, 0.5 mM) for 16 h with or without cimifugin (Cim, 40 μM) pretreatment for 2 h. Intracellular triglyceride (TG) was measured as described in Material and Methods. All values are denoted as means ± SD from at least three independent batches of cells. ∗ and # reflect statistical difference (P < 0.05) compared with the control group and palmitate treatment group, respectively. Figure S2: the source and culture method of HepG2 hepatocytes were same as those of Figure S1. HepG2 hepatocytes were treated with palmitic acid (PA, 0.5 mM) for 16 h with or without cimifugin (Cim, 40 μM) pretreatment for 2 h. SIRT1 and TLR4 expressions were measured by immunoblotting. ∗ and # reflect statistical difference (P < 0.05) compared with the control group and palmitate treatment group, respectively. Figure S3: the source and culture method of HepG2 hepatocytes were same as those of Figure S1. HepG2 hepatocytes were treated with oleic acid (OA, 0.5 mM) for 16 h with or without cimifugin (Cim, 40 μM) pretreatment for 2 h. Intracellular triglyceride (TG) was measured as described in Material and Methods. ∗ and # reflect statistical difference (P < 0.05) compared with the control group and palmitate treatment group, respectively. Figure S4: the source and culture method of HepG2 hepatocytes were same as those of Figure S1. HepG2 hepatocytes were treated with palmitic acid (PA, 0.5 mM) for 16 h with or without cimifugin (Cim, 40 μM) pretreatment for 2 h. Intracellular triglyceride (TG) was measured as described in Material and Methods. ∗ and # reflect statistical difference (P < 0.05) compared with the c [file 4557532.f1.pdf]

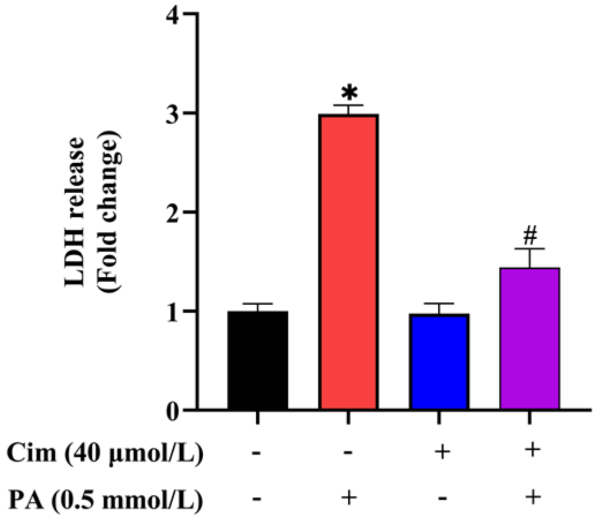

Figure S1 HepG2，a widely used human hepatoma cell line, was obtained from American Type Culture Collection (ATCC, Manassas, VA). HepG2 hepatocytes were cultured in Dulbecco's Modified Eagle Medium (DMEM, Gibco) containing 10% (v/v) fetal bovine serum (FBS, Biological Industries) at 37 °C in a humidified atmosphere of 5% CO_2 and 95% air. HepG2 hepatocytes were treated with palmitic acid (PA, 0.5 mM) for 16 h with or without cimifugin (Cim, 40 μM) pretreatment for 2 h. Intracellular triglyceride (TG) was measured as described in the Methods. All values are denoted as means ± SD from at least three independent batches of cells. * and # reflect statistical difference (*P* < 0.05) compared with control group and palmitate treatment group respectively.

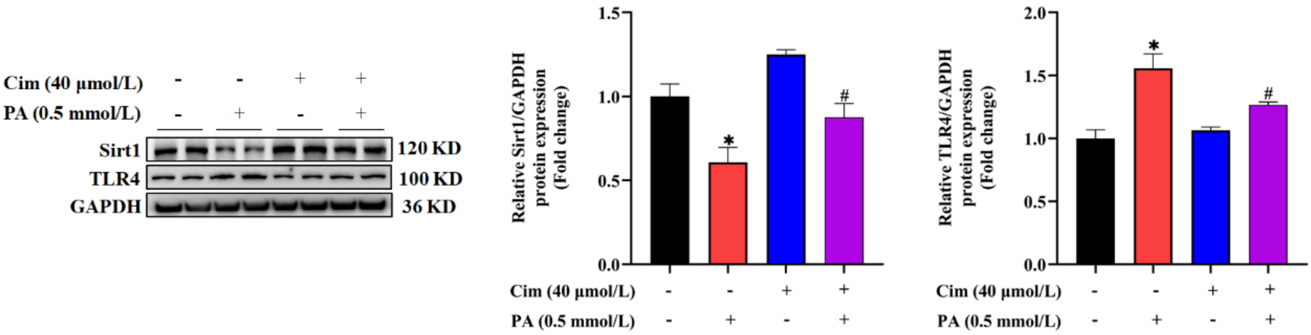

Figure S2 The source and culture method of HepG2 hepatocytes were same as those of Figure S1. HepG2 hepatocytes were treated with palmitic acid (PA, 0.5 mM) for 16 h with or without cimifugin (Cim, 40 μM) pretreatment for 2 h. SIRT1 and TLR4 expressions were measured by immunoblotting. * and # reflect statistical difference ($P < 0.05$) compared with control group and palmitate treatment group respectively.

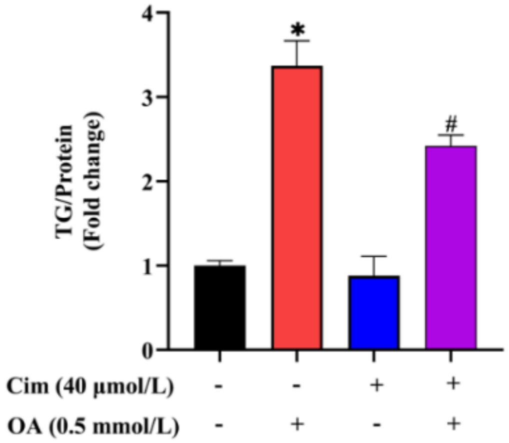

Figure S3 The source and culture method of HepG2 hepatocytes were same as those of Figure S1. HepG2 hepatocytes were treated with oleic acid (OA, 0.5 mM) for 16 h with or without cimifugin (Cim, 40 μM) pretreatment for 2 h. Intracellular triglyceride (TG) was measured as described in the Methods. * and # reflect statistical difference ($P < 0.05$) compared with control group and palmitate treatment group respectively.

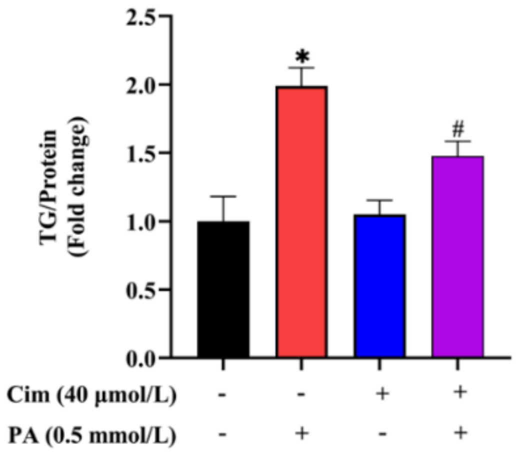

Figure S4 The source and culture method of HepG2 hepatocytes were same as those of Figure S1. HepG2 hepatocytes were treated with palmitic acid (PA, 0.5 mM) for 16 h with or without cimifugin (Cim, 40 μM) pretreatment for 2 h. Intracellular triglyceride (TG) was measured as described in the Methods. * and # reflect statistical difference (*P* < 0.05) compared with control group and palmitate treatment group respectively.

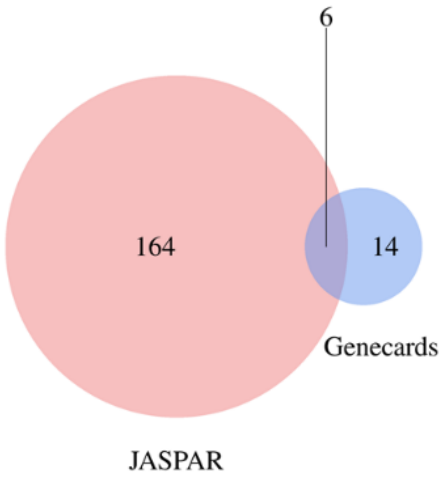

Figure S5 The TLR4 promoter sequence (NC_000070.7, -2000~100) were searched in the websites including JASPAR (https://jaspar.genereg.net/) and Genecards data base (http://www.genecards.org), and the co-existing nuclear factors were obtained in these data bases.
